# Proactive Treatment of Canine Allergic Otitis Externa With Hydrocortisone Aceponate: A Retrospective Observational Study

**DOI:** 10.1002/vms3.71276

**Published:** 2026-10-21

**Authors:** Elisa Maina, Jasmine Chantale Rigert, Carlotta Filippini, Regula Cao, Ana Oliveira

**Affiliations:** ^1^ Division of Clinical Dermatology Department of Clinical Veterinary Medicine Vetsuisse Faculty University of Bern Bern Switzerland; ^2^ Faculty of Veterinary Medicine CIISA – Centre for Interdisciplinary Research in Animal Health University of Lisbon Lisbon Portugal; ^3^ Associate Laboratory for Animal and Veterinary Sciences (AL4AnimalS) Lisbon Portugal

**Keywords:** allergy, dogs, hydrocortisone aceponate, otitis externa, recurrence

## Abstract

**Background:**

Allergic otitis externa is common in dogs and recurrence may lead to repeated consultations and antimicrobial use. Long‐term data on proactive topical corticosteroid maintenance are limited.

**Objectives:**

To describe 12‐month relapse frequency in dogs with allergic otitis externa receiving proactive hydrocortisone aceponate maintenance therapy and compare consecutive‐day and non‐consecutive‐day schedules.

**Methods:**

Medical records of client‐owned dogs prescribed hydrocortisone aceponate ear spray were retrospectively reviewed. Maintenance started after clinical and cytological remission. Only dogs with confirmed continuation, stable concomitant allergy management and complete 12‐month outcome data were included. Relapse was defined as recurrent otitis externa requiring veterinary examination, cytology and additional otic treatment. Dogs were grouped by schedule: Protocol 1, two consecutive days; and Protocol 2, two non‐consecutive days weekly. Outcomes were compared using Fisher exact and Mann–Whitney *U* tests.

**Results:**

Seventy‐one dogs were included; 36 in Protocol 1 and 35 received Protocol 2. During 12 months, 50/71 dogs (70.4%) had no relapse, 12/71 (16.9%) had one relapse and 9/71 (12.7%) had multiple relapses. At least one relapse occurred in 9/36 dogs (25.0%) in Protocol 1 and 12/35 (34.3%) receiving Protocol 2 (*p* = 0.443). Multiple relapses occurred in 2/36 (5.6%) and 7/35 (20.0%) dogs, respectively (*p* = 0.085). Relapse counts did not differ significantly between protocols (*p* = 0.290).

**Conclusions:**

Most dogs had no relapse or only one relapse during proactive hydrocortisone aceponate maintenance. No statistically significant difference was detected between schedules, but equivalence or non‐inferiority cannot be concluded.

## Graphical abstract

In 71 dogs with allergic otitis externa receiving proactive hydrocortisone aceponate maintenance therapy after clinical and cytological remission, 70.4% had no relapse and 87.3% had no relapse or only one relapse during a fixed 12‐month observation period. No statistically significant difference was detected between maintenance schedules; however, the study was not designed to demonstrate equivalence or non‐inferiority between protocols.

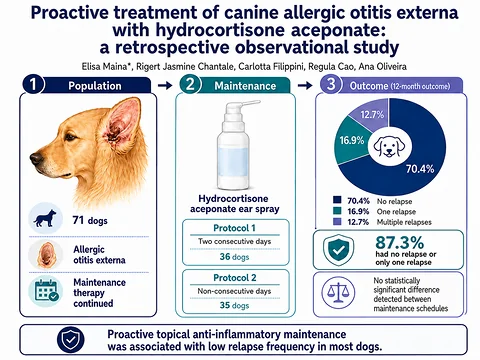

## 1 Introduction

Otitis externa is a common and clinically important condition in dogs. In allergic dogs, inflammation of the external ear canal may occur as a primary manifestation of disease, may accompany more generalised cutaneous signs, or may persist after apparent control of pruritus elsewhere. Once inflammation is established, changes in the ear canal microenvironment favour cerumen accumulation and secondary yeast or bacterial overgrowth, and repeated episodes may promote chronic structural changes that further complicate long‐term control (Nuttall 2023; Rosser 2004).

For this reason, management of allergic otitis externa is not limited to treatment of active infection. Control of the underlying inflammatory component is central to reducing recurrence, limiting repeated antimicrobial use and preventing progression towards chronic ear canal changes. This concept is consistent with the wider approach to canine atopic dermatitis, in which proactive anti‐inflammatory treatment after remission may reduce allergic flares rather than waiting for relapse to occur (Lourenço et al. 2016; Olivry et al. 2015).

Hydrocortisone aceponate is a non‐halogenated diester glucocorticoid developed for topical use. Its pharmacological profile is intended to provide local anti‐inflammatory activity with limited systemic exposure, and previous studies in canine atopic dermatitis reported clinical efficacy without detectable adrenal suppression during short‐term use (Nam et al. 2012; Nuttall et al. 2009). More recently, hydrocortisone aceponate ear spray has been evaluated in dogs with erythemato‐ceruminous otitis externa, where short‐course treatment improved clinical signs similarly to an antimicrobial‐containing combination product (Rigaut et al. 2024). Long‐term retrospective data also support its use as part of reactive and proactive management of chronic or recurrent non‐infectious otitis externa (Lorente‐Mendez and Alonso‐Miguel 2025).

In clinical practice, however, maintenance schedules are not uniform. Some clinicians prescribe two consecutive treatment days each week, whereas others use non‐consecutive days. The relative importance of this difference is unknown. The primary objective of the present retrospective multicentre study was to describe relapse frequency over a fixed 12‐month observation period in dogs with allergic otitis externa receiving proactive hydrocortisone aceponate maintenance therapy after clinical and cytological remission. A secondary objective was to compare relapse frequency between consecutive‐day and non‐consecutive‐day maintenance protocols, without assuming equivalence between schedules.

## 2 Materials and Methods

### 2.1 Study Design and Reporting

This was a retrospective multicentre observational study of client‐owned dogs with allergic otitis externa receiving proactive topical hydrocortisone aceponate maintenance therapy over a fixed 12‐month observation period. The design and reporting were guided by STROBE recommendations for observational studies (von Elm et al. 2007).

The unit of analysis was the dog. When both ears were affected, the dog was classified according to the more severe or clinically relevant otitis phenotype recorded before maintenance therapy.

### 2.2 Case Selection

Medical records were reviewed to identify dogs diagnosed with allergic otitis externa and prescribed proactive maintenance therapy with hydrocortisone aceponate 0.584 mg/mL ear spray solution (Cortotic; Virbac, Carros, France). Allergic otitis externa was diagnosed by the attending clinician on the basis of compatible history, clinical, cytological and otoscopic findings, exclusion or treatment of secondary infection, and the presence or suspicion of allergic skin disease, including atopic dermatitis, food allergy or both. The diagnosis required exclusion of other primary causes of otitis, including ectoparasites and foreign bodies where clinically relevant, together with control of secondary bacterial or yeast infection. Flea infestation and flea‐allergy dermatitis were excluded or controlled. Food allergy was assessed when clinically indicated with an elimination diet and oral food challenge, and canine atopic dermatitis was diagnosed using accepted clinical criteria after exclusion of relevant differential diagnoses.

Only dogs for which treatment continuation was confirmed by owner telephone follow‐up and for which complete information for a fixed 12‐month observation period was available were included in the final analysis. Maintenance therapy was started only after clinical and cytological remission of the active otitis episode had been achieved. Concomitant allergy management, including systemic antipruritic or anti‐inflammatory treatment, allergen‐specific immunotherapy and diet, had to remain unchanged during the 12‐month observation period. Dogs were excluded if treatment had not been continued, if continuation could not be confirmed, if updated relapse information could not be obtained, or if follow‐up was shorter than 12 months. Dogs were also excluded if the maintenance schedule was changed, if hydrocortisone aceponate was not administered as prescribed, if concomitant allergy treatment or diet was initiated, discontinued or changed during follow‐up, or if complete 12‐month outcome data were not available.

### 2.3 Data Collection

Data extracted from medical records included signalment, diagnosis, laterality of otitis before maintenance therapy, clinical type of otitis, cytological findings, maintenance protocol, concomitant allergy management when recorded, follow‐up information and number of relapses during the study period. Owner telephone follow‐up was performed to confirm whether the dog had continued maintenance treatment and to update relapse information. When no response was obtained, the dog was excluded from the final analysis. Therefore, all 71 dogs included in the final cohort had confirmed treatment continuation, updated relapse information and complete 12‐month follow‐up.

### 2.4 Treatment Protocols

All included dogs received proactive hydrocortisone aceponate maintenance therapy. Dogs were classified into two groups according to the prescribed schedule. Protocol 1 consisted of application on two consecutive days. Protocol 2 consisted of application on two non‐consecutive days per week, separated by at least two treatment‐free days. Treatment allocation was not randomised and reflected the attending clinician's judgement and routine practice.

During maintenance, clinically stable dogs received a chlorhexidine‐based ear cleaner once weekly, 24 h before hydrocortisone aceponate application. This ear‐cleaning schedule was used once weekly irrespective of whether hydrocortisone aceponate was applied on two consecutive or two non‐consecutive days.

Treatment used to achieve remission before entry into the maintenance period, and rescue treatment used in case of relapse, were selected according to otoscopic findings and otic cytology. *Malassezia* overgrowth was treated with a topical combination of miconazole nitrate, polymyxin B sulphate and prednisolone acetate, eight drops twice daily for 14 days, combined with a chlorhexidine‐based ear cleaner once daily for 14 days. Coccoid bacterial infection, defined cytologically by cocci associated with neutrophilic inflammation and/or intracellular bacteria, was treated with a topical combination of hydrocortisone aceponate, miconazole nitrate and gentamicin sulphate, one pump once daily for 14 days, combined with a chlorhexidine/Tris‐EDTA ear solution once daily for 14 days. Rod‐shaped bacterial infection and mixed infection (rods and cocci) were treated with a topical combination of marbofloxacin, clotrimazole and dexamethasone acetate, eight drops once daily for 14 days, combined with a chlorhexidine/Tris‐EDTA ear solution once daily for 14 days. When *Pseudomonas* spp. infection was diagnosed, an N‐acetylcysteine/Tris‐EDTA solution, 2 mL once daily, was applied 30 min before a topical enrofloxacin/dexamethasone/sodium chloride solution once daily; these cases also received oral prednisolone at 1 mg/kg once daily for 14 days. Dogs requiring a different rescue regimen or a change in maintenance or allergy management were not retained in the final analysis.

### 2.5 Outcome Measures

The primary outcome was the occurrence of at least one relapse during maintenance therapy within the 12‐month observation period. Relapse was defined as a recurrent episode of otitis externa requiring veterinary examination, cytological confirmation and additional otic treatment. Secondary outcomes were occurrence of multiple relapses, defined as two or more relapses, and total relapse count during the observation period. Because all dogs were observed for exactly 12 months, the total relapse count per dog was equivalent to the relapse rate per dog‐year.

### 2.6 Statistical Analysis

Descriptive statistics were used to summarise population characteristics and treatment outcomes. Continuous variables were reported as median and range. Categorical variables were reported as number and percentage. Signalment and baseline otitis characteristics were compared between treatment protocols. Continuous variables were compared with Mann–Whitney *U* tests. Categorical variables were compared using Fisher exact tests for 2 × 2 tables or chi‐square tests for larger contingency tables. The proportions of dogs with at least one relapse and with multiple relapses were compared between protocols using Fisher exact tests. Relapse counts were compared using a Mann–Whitney *U* test. Relative risks and 95% confidence intervals were calculated for Protocol 2 compared with Protocol 1. Statistical significance was set at *p* < 0.05. Because the comparison between protocols was secondary and exploratory, results were interpreted with emphasis on effect size, confidence intervals and clinical plausibility rather than on *p* values alone. No non‐inferiority or equivalence margin was prespecified, and the study was not designed or powered as a non‐inferiority or equivalence trial.

## 3 Results

### 3.1 Study Population

Medical records of 104 dogs prescribed hydrocortisone aceponate ear spray for proactive management of allergic otitis externa were screened. Seventy‐one dogs met the final inclusion criteria. Thirty‐three dogs were excluded because treatment was not started or not continued for the required period, the maintenance schedule or concomitant allergy management changed, owner follow‐up could not confirm the required information, or complete 12‐month outcome data were not available. All dogs received hydrocortisone aceponate ear spray. All included dogs had 12 months of follow‐up, and continuation of the prescribed maintenance protocol was confirmed by owner telephone follow‐up. Thirty‐six dogs received Protocol 1 and 35 dogs received Protocol 2. Study population and treatment protocol distribution are summarised in Table 1. Age differed between groups: dogs receiving Protocol 1 had a median age of 5 years (range, 1–14), whereas dogs receiving Protocol 2 had a median age of 7 years (range, 1–15; *p* = 0.041). Body weight and sex distribution did not differ significantly between groups (*p* = 0.324 and *p* = 0.196, respectively; Table 1). Forty‐six breeds were represented. The most frequent breeds were French Bulldog (8/71), English/American Cocker Spaniel (8/71), Labrador Retriever (6/71), Golden Retriever (3/71) and Maltese (3/71).

**TABLE 1:** Study population and comparison of signalment data between treatment protocols.

Variable	Overall cohort (*n* = 71)	Protocol 1 (*n* = 36)	Protocol 2 (*n* = 35)	*p* value
Age, years, median (range)	6 (1–15)	5 (1–14)	7 (1–15)	0.041
Body weight, kg, median (range)	17.5 (3–64); *n* = 60	15.5 (3–64); *n* = 28	18.5 (4–44); *n* = 32	0.324
Intact male	21 (29.6%)	12 (33.3%)	9 (25.7%)	
Neutered male	17 (23.9%)	10 (27.8%)	7 (20.0%)	
Intact female	7 (9.9%)	5 (13.9%)	2 (5.7%)	
Spayed female	26 (36.6%)	9 (25.0%)	17 (48.6%)	0.196
Protocol distribution	71 (100%)	36 (50.7%)	35 (49.3%)	—

*Note*: Data are presented as *n* (%) unless otherwise stated. *p* values are from Mann–Whitney *U* tests for continuous variables and chi‐square testing for sex distribution.

### 3.2 Baseline Otitis Characteristics

Before maintenance therapy, otitis externa was bilateral in 55/71 dogs (77.5%) and unilateral in 16/71 dogs (22.5%). The most common baseline clinical type was ceruminous otitis, followed by erythematous, purulent and ulcerative otitis. Baseline cytology most commonly identified *Malassezia* spp.; no organisms were recorded in 17/71 dogs (23.9%). Baseline otitis characteristics are summarised in Table 2. Baseline laterality, clinical type, cytological category and amount score did not differ significantly between protocols (Table 2).

**TABLE 2:** Baseline otitis characteristics before induction of remission and therapy and comparison between protocols.

Baseline characteristic	Overall (*n* = 71)	Protocol 1 (*n* = 36)	Protocol 2 (*n* = 35)	*p* value
Bilateral otitis before maintenance therapy	55/71 (77.5%)	27/36 (75.0%)	28/35 (80.0%)	0.778
Unilateral otitis before maintenance therapy	16/71 (22.5%)	9/36 (25.0%)	7/35 (20.0%)	
Erythematous otitis	20/71 (28.2%)	7/36 (19.4%)	13/35 (37.1%)	
Ceruminous otitis	39/71 (54.9%)	23/36 (63.9%)	16/35 (45.7%)	
Purulent otitis	10/71 (14.1%)	5/36 (13.9%)	5/35 (14.3%)	
Ulcerative otitis	2/71 (2.8%)	1/36 (2.8%)	1/35 (2.9%)	0.385
Baseline cytology: *Malassezia* spp.	32/71 (45.1%)	21/36 (58.3%)	11/35 (31.4%)	
Baseline cytology: no organisms recorded	17/71 (23.9%)	9/36 (25.0%)	8/35 (22.9%)	
Baseline cytology: mixed infection	8/71 (11.3%)	2/36 (5.6%)	6/35 (17.1%)	
Baseline cytology: cocci	8/71 (11.3%)	2/36 (5.6%)	6/35 (17.1%)	
Baseline cytology: rods	4/71 (5.6%)	2/36 (5.6%)	2/35 (5.7%)	
Different cytology between ears	2/71 (2.8%)	0/36 (0%)	2/35 (5.7%)	0.102
Baseline amount score, median (range)	1 (0–5)	2 (0–5)	1 (0–5)	0.732

*Note*: For dogs with different clinical type or cytological findings between ears, the more severe clinical type was used for classification where applicable. *p* values are from Fisher exact testing for laterality, chi‐square testing for clinical type and cytological category, and Mann–Whitney *U* testing for amount score.

### 3.3 Overall Relapse Outcome

Over the 12‐month observation period, 50/71 dogs (70.4%; 95% CI, 58.4–80.7%) had no relapse and 21/71 dogs (29.6%; 95% CI, 19.3–41.6%) had at least one relapse. Twelve dogs (16.9%; 95% CI, 9.0–27.7%) had one relapse, whereas nine dogs (12.7%; 95% CI, 6.0–22.7%) had multiple relapses. Overall relapse outcomes and comparisons between protocols are summarised in Table 3. The total relapse count was 39 over 71 dog‐years, corresponding to 0.55 relapses/dog‐year.

**TABLE 3:** Relapse outcomes overall and according to maintenance protocol during the fixed 12‐month observation period.

Outcome	Overall (*n* = 71)	Protocol 1 (*n* = 36)	Protocol 2 (*n* = 35)	Effect estimate	*p* value
No relapse	50 (70.4%)	27 (75.0%)	23 (65.7%)	—	—
At least one relapse	21 (29.6%)	9 (25.0%)	12 (34.3%)	RR 1.37 (95% CI, 0.66–2.84)	0.443
One relapse only	12 (16.9%)	7 (19.4%)	5 (14.3%)	—	—
Multiple relapses (≥2)	9 (12.7%)	2 (5.6%)	7 (20.0%)	RR 3.60 (95% CI, 0.80–16.15)	0.085
Total relapse count	39	14	25	Not estimated	0.290
Relapse rate, relapses/dog‐year	0.55	0.39	0.71	Not estimated	0.290
Median relapse count (range)	0 (0–5)	0 (0–4)	0 (0–5)	Not estimated	0.290

*Note*: Protocol 1 = application on two consecutive days; Protocol 2 = application on non‐consecutive days. RR = relative risk for Protocol 2 compared with Protocol 1; CI = confidence interval. Because all dogs were observed for 12 months, relapse count per dog is equivalent to relapses per dog‐year. Fisher exact tests were used for categorical outcomes and the Mann–Whitney *U* test for relapse counts.

### 3.4 Comparison Between Maintenance Protocols

At least one relapse occurred in 9/36 dogs (25.0%; 95% CI, 12.1–42.2%) receiving Protocol 1 and in 12/35 dogs (34.3%; 95% CI, 19.1–52.2%) receiving Protocol 2. This difference was not statistically significant (relative risk for Protocol 2 compared with Protocol 1, 1.37; 95% CI, 0.66–2.84; *p* = 0.443). Multiple relapses occurred in 2/36 dogs (5.6%; 95% CI, 0.7–18.7%) receiving Protocol 1 and 7/35 dogs (20.0%; 95% CI, 8.4–36.9%) receiving Protocol 2. This numerical difference did not reach statistical significance (relative risk, 3.60; 95% CI, 0.80–16.15; *p* = 0.085). Total relapse count was 14 in Protocol 1 and 25 in Protocol 2. Because each dog contributed one year of observation, relapse rates were 0.39 relapses/dog‐year for Protocol 1 and 0.71 relapses/dog‐year for Protocol 2. Median relapse count was 0 in both groups, and relapse counts did not differ significantly between protocols (*p* = 0.290; Table 3).

## 4 Discussion

The present study describes relapse frequency during a 12‐month observation period in dogs with allergic otitis externa that entered proactive hydrocortisone aceponate maintenance therapy after clinical and cytological remission. In this verified cohort, 70.4% of dogs had no relapse and a further 16.9% had only one relapse. These findings are broadly consistent with the recent retrospective study by Lorente‐Méndez and Alonso‐Miguel, in which hydrocortisone aceponate was used for the reactive and proactive management of non‐infectious chronic or recurrent otitis externa, and 79.1% of treated ears remained recurrence‐free over a mean follow‐up of 202.6 days (Lorente‐Mendez and Alonso‐Miguel 2025). Direct comparison is limited because that study analysed ears rather than dogs, included chronic or recurrent non‐infectious otitis externa rather than allergic otitis externa specifically, used severity‐adapted treatment schedules, and had a variable follow‐up period. By contrast, the present study used the dog as the unit of analysis, required clinical and cytological remission before maintenance therapy, and assessed relapse over a fixed 12‐month observation period. Nevertheless, both studies support the use of sustained topical anti‐inflammatory maintenance therapy for the prevention of relapses of recurrent allergic otitis externa.

Dogs treated on two consecutive days had numerically fewer relapses, particularly multiple relapses, than dogs treated on non‐consecutive days. However, this finding must be interpreted with caution, as no statistically significant difference was detected between the two maintenance schedules. The study was not designed or powered as a non‐inferiority or equivalence trial, and the small number of dogs and relapse events limits the ability to detect a true difference between schedules. The lower relapse frequency observed in the consecutive‐day group should be considered hypothesis‐generating and requires evaluation in a prospective controlled study with adequate sample size.

The comparison between schedules was also limited by the observational nature of the study. Treatment allocation was not randomised and reflected routine clinical practice. Although baseline characteristics were compared between groups, unmeasured differences in previous relapse pattern, ear canal changes, owner adherence or clinician preference may have influenced the choice of maintenance schedule. In addition, dogs in Protocol 2 were older than dogs in Protocol 1, and age cannot be excluded as a potential confounding factor. Therefore, the protocol comparison should be interpreted as exploratory rather than confirmatory.

The results should not be interpreted as evidence that maintenance therapy alone controls all aspects of allergic otitis externa. Proactive topical treatment targets the inflammatory component of the ear canal, but allergic disease remains multifactorial. Allergen avoidance where possible, control of ectoparasites, management of food allergy when present, appropriate treatment of secondary infection, ear cleaning when indicated and systemic anti‐inflammatory or antipruritic therapy may all influence outcome. Previous work in canine atopic dermatitis has shown that hydrocortisone aceponate can reduce skin lesions and pruritus (Nam et al. 2012; Nuttall et al. 2009; Nuttall et al. 2012), and combination with systemic treatment may help prevent deterioration when systemic therapy is tapered (Takahashi et al. 2021).

The broader proactive‐treatment concept is also supported by Lourenço et al. (2016), in which intermittent hydrocortisone aceponate spray prolonged time to relapse in canine atopic dermatitis, although that study was prospective, placebo‐controlled and focused on cutaneous lesions rather than otitis externa.

In the present study, dogs were retained in the final analysis only if concomitant allergy management remained stable during the 12‐month observation period. This included systemic antipruritic or anti‐inflammatory treatment, allergen‐specific immunotherapy and diet, when applicable. This criterion was intended to reduce the effect of major therapeutic changes on otitis relapse frequency. Nevertheless, concurrent allergy treatments were not randomised or standardised, and their possible influence on relapse risk cannot be excluded.

By including only dogs with confirmed continuation of the prescribed maintenance protocol, stable concomitant allergy management and complete 12‐month outcome information, the present analysis gives a clearer estimate of relapse frequency among dogs that actually received the intended maintenance regimen. However, this choice also introduces selection bias. Dogs that stopped treatment, used the product incorrectly, changed allergy treatment, lacked complete records or could not be contacted were excluded. Dogs that discontinued treatment because of perceived lack of efficacy, or because they were stable and owners elected to stop treatment, were therefore not represented in the final cohort. The results should consequently be interpreted as outcomes in a verified adherent cohort, not as an intention‐to‐treat estimate of all dogs initially prescribed hydrocortisone aceponate maintenance therapy.

Relapse was defined more strictly than owner‐reported recurrence alone, requiring veterinary examination, otic cytology and additional otic treatment. However, retrospective relapse assessment remains vulnerable to recall and recording bias. Minor episodes managed by owners without veterinary examination, or episodes treated elsewhere without complete documentation, may have been missed. Exact dates of relapse were not consistently available, so time‐to‐first‐relapse analysis could not be performed. This limitation is partly mitigated by the fixed 12‐month observation period, which allowed relapse frequency and relapse rate per dog‐year to be compared between groups, but it prevents comparison of relapse timing. Adherence was confirmed by owner report but was not objectively measured. Finally, the study had no untreated control group; therefore, it cannot prove that hydrocortisone aceponate maintenance therapy caused the low relapse frequency, only that relapse frequency was low in dogs that continued the intended regimen under stable management conditions.

## 5 Conclusion

In this retrospective field cohort of dogs with allergic otitis externa that entered proactive hydrocortisone aceponate maintenance therapy after clinical and cytological remission, most dogs had no relapse or only one relapse during a fixed 12‐month observation period. No statistically significant difference was detected between consecutive‐day and non‐consecutive‐day maintenance protocols; however, the study was not designed or powered to demonstrate equivalence or non‐inferiority between schedules. These findings support further prospective evaluation of proactive topical anti‐inflammatory maintenance therapy as part of long‐term management of allergic otitis externa, including adequately powered comparison of maintenance schedules and standardised assessment of relapse timing.

## Author Contributions

**Elisa Maina**: Conceptualisation; methodology; investigation; formal analysis; supervision; project administration; writing – original draft; writing – review and editing. **Rigert Jasmine Chantale**: Investigation; data curation; writing – review and editing. **Carlotta Filippini**: Investigation; data curation; writing – review and editing. **Regula Cao**: Investigation; data curation; validation; writing – review and editing. **Ana Oliveira**: Validation; writing – review and editing.

## Funding

The authors have nothing to report.

## Ethics Statement

The authors confirm that the ethical policies of Veterinary Medicine and Science have been adhered to. This was a retrospective review of clinical records from client‐owned dogs treated as part of routine veterinary care with a commercially licensed veterinary medicinal product authorised for otic use. No procedures were performed for research purposes and formal ethical approval was not required under the applicable institutional and national regulations.

## Patient/Client Consent Statement

Informed client consent was obtained for diagnostic and therapeutic procedures performed as part of routine veterinary care. Owner telephone follow‐up was performed to confirm treatment continuation and clinical outcome. Data were anonymised before analysis.

## Conflicts of Interest

The authors declare no conflicts of interest.

## Data Availability

The data that support the findings of this study are not publicly available because they contain confidential clinical information from client‐owned animals. Anonymised data may be made available from the corresponding author upon reasonable request and subject to applicable institutional and data‐protection requirements.

## References

[vms371276-bib-0001] Lorente‐Méndez, C. , and D. Alonso‐Miguel . 2025. “Hydrocortisone Aceponate for Chronic Otitis: Long‐Term Efficacy.” Veterinary Dermatology 36: 870–878. 10.1111/vde.70008 PMC12590094 40757500

[vms371276-bib-0002] Lourenço, A. M. , V. Schmidt , B. São Braz , et al. 2016. “Efficacy of Proactive Long‐Term Maintenance Therapy of Canine Atopic Dermatitis With 0.0584% Hydrocortisone Aceponate Spray: A Double‐Blind Placebo Controlled Pilot Study.” Veterinary Dermatology 27, no. 2: 88. 10.1111/vde.12285 26804943

[vms371276-bib-0003] Nam, E. H. , S. H. Park , J. i. Y. Jung , et al. 2012. “Evaluation of the Effect of a 0.0584% Hydrocortisone Aceponate Spray on Clinical Signs and Skin Barrier Function in Dogs With Atopic Dermatitis.” Journal of Veterinary Science 13, no. 2: 187. 10.4142/jvs.2012.13.2.187 PMC3386344 22705741

[vms371276-bib-0004] Nuttall, T. 2023. “Managing Recurrent Otitis Externa in Dogs: What Have We Learned and What Can We Do Better?” Journal of the American Veterinary Medical Association 261, no. S1: S10–S22. 10.2460/javma.23.01.0002 37019436

[vms371276-bib-0005] Nuttall, T. , R. Mueller , E. Bensignor , et al. 2009. “Efficacy of a 0.0584% Hydrocortisone Aceponate Spray in the Management of Canine Atopic Dermatitis: A Randomised, Double Blind, Placebo‐Controlled Trial.” Veterinary Dermatology 20, no. 3: 191–198. 10.1111/j.1365-3164.2009.00756.x 19374721

[vms371276-bib-0006] Nuttall, T. J. , N. A. Mcewan , E. Bensignor , L. Cornegliani , C. Löwenstein , and C. A. Rème . 2012. “Comparable Efficacy of a Topical 0.0584% Hydrocortisone Aceponate Spray and Oral Ciclosporin in Treating Canine Atopic Dermatitis.” Veterinary Dermatology 23, no. 1: 4. 10.1111/j.1365-3164.2011.00992.x 21718368

[vms371276-bib-0007] Olivry, T. , D. J. Deboer , C. Favrot , et al. 2015. “Treatment of Canine Atopic Dermatitis: 2015 Updated Guidelines From the International Committee on Allergic Diseases of Animals.” BMC Veterinary Research 11: 210. 10.1186/s12917-015-0514-6 PMC4537558 26276051

[vms371276-bib-0008] Rigaut, D. , P. Briantais , P. Jasmin , and A. Bidaud . 2024. “Efficacy and Safety of a Hydrocortisone Aceponate‐Containing Ear Spray Solution in Dogs With Erythemato‐Ceruminous Otitis Externa: A Randomised, Multicentric, Single‐Blinded, Controlled Trial.” Veterinary Dermatology 35, no. 2: 197–206. 10.1111/vde.13224 38093088

[vms371276-bib-0009] Rosser, E. J. 2004. “Causes of Otitis Externa.” Veterinary Clinics of North America: Small Animal Practice 34, no. 2: 459–468. 10.1016/j.cvsm.2003.10.006 15062619

[vms371276-bib-0010] Takahashi, J. , S. Kanda , I. Imanishi , et al. 2021. “Efficacy and Safety of 0.0584% Hydrocortisone Aceponate Topical Spray and Systemic Oclacitinib Combination Therapy in Dogs With Atopic Dermatitis: A Randomized, Double‐Blinded, Placebo‐Controlled Trial.” Veterinary Dermatology 32, no. 2: 119. 10.1111/vde.12909 33185330

[vms371276-bib-0011] von Elm, E. , D. G. Altman , M. Egger , et al. 2007. “The Strengthening the Reporting of Observational Studies in Epidemiology (STROBE) Statement: Guidelines for Reporting Observational Studies.” Lancet 370, no. 9596: 1453–1457. 10.1016/S0140-6736(07)61602-X 18064739

